# Live monitoring of plant redox and energy physiology with genetically encoded biosensors

**DOI:** 10.1093/plphys/kiab019

**Published:** 2021-01-28

**Authors:** Stefanie J Müller-Schüssele, Markus Schwarzländer, Andreas J Meyer

**Affiliations:** 1 Institute of Crop Science and Resource Conservation (INRES), University of Bonn, Friedrich-Ebert-Allee 144, D-53113 Bonn, Germany; 2 Institute of Plant Biology and Biotechnology, University of Münster, Schlossplatz 8, D-48143 Münster, Germany

## Abstract

Genetically encoded biosensors pave the way for understanding plant redox dynamics and energy metabolism on cellular and subcellular levels.

**ADVANCES**

- Methodological advances in fluorescent protein-based *in vivo* biosensing have been instrumental for several paradigm shifts in our understanding of cell physiology, metabolism and signaling.
- An increasing number of genetically encoded biosensors has been used to dissect the dynamics of several distinct redox couples and energy physiology in plants.
- *In vivo* monitoring using biosensors has pioneered the simultaneous read-out of different physiological parameters in different subcellular locations by parallelized plate reader-based, multiwell fluorimetry, or expression strategies for multiple sensors in parallel.
- Sensing dynamic changes in hydrogen peroxide levels is possible with sensors of the HyPer family, or roGFP fusion variants with a thiol peroxidase.
- Peredox and SoNar family sensors enable direct visualization of NADH/NAD^+^, while iNAP family sensors respond to NADPH concentration in plants.
- Sensor variants with different sensitivity ranges enable use of the most appropriate variant for the specific *in vivo* environment or experimental scope.

## Integration of redox regulation into plant energy metabolism

In eukaryotic cells, cell compartments fulfill different yet complementary functions, while they are linked by the cellular metabolic network. In addition, plants possess photosynthetic and non-photosynthetic tissues, which most visibly differ by the types of plastids they contain. In photosynthetic tissues, plants can shift flexibly from photo-autotrophic to heterotrophic metabolism, depending on the presence of light, which increases the challenge of maintaining metabolic control. During active photosynthesis, chloroplasts produce ATP, reduce equivalents in the light reactions and fix carbon in the Calvin–Benson–Bassham (CBB) cycle. The discovery of thioredoxins (TRXs) as light-dependent Cys-redox switch operators for multiple enzymes in carbon assimilation by Wolosiuk and Buchanan (1977) established a hotspot of Cys-based redox regulation, which is directly connected to the availability of photosynthesis-derived reductant. Maintaining photosynthetic efficiency additionally relies on active adjustment of the ATP:NADPH ratio by mitochondria acting as electron sinks, as well as the recycling of 2-phosphoglycolate during photorespiration including multiple reaction steps in peroxisomes and mitochondria. In the dark and heterotrophic tissues, mitochondria make a major contribution to energy metabolism by respiration.

Generally, pools of metabolites and reducing equivalents are separated between compartments and selectively linked via specific transport across membranes. While stromal and cytosolic pools of reduced glutathione (GSH) are connected by chloroquine-resistance transporter-like transporters (CLTs; Maughan et al., 2010), the oxidized form of glutathione, glutathione disulfide (GSSG), cannot be transported and requires local reducing systems in both the cytosol and the stroma (Marty et al., 2009; Marty et al., 2019). Similarly, NAD^+^ can be transported across the membranes of the plastids, mitochondria, and peroxisomes (van Roermund et al., 2016; Gakière et al., 2018; Souza Chaves et al., 2019; Feitosa-Araujo et al., 2020), while NADH is not directly transported across membranes necessitating the indirect transport of reducing equivalents via metabolite shuttles (Hoefnagel et al., 1998; Selinski and Scheibe, 2018). Hydrogen peroxide (H_2_O_2_) transport across specific membranes may be facilitated by certain aquaporins (Smirnoff and Arnaud, 2019).

Thus, intracellular boundaries with selective connectivity are necessary for metabolism of eukaryotic cells with specific membrane contact sites between different compartments additionally emerging as local exchange and signaling hubs (Pérez-Sancho et al., 2016; Baillie et al., 2020). Reactive oxygen species (ROS) serve as important signaling components in plant development and stress responses (Waszczak et al., 2018; Smirnoff and Arnaud, 2019). ROS can be specifically and locally generated by enzymes, or are formed under stress conditions, especially in the chloroplasts and mitochondria, which harbor electron transport chains that can release specific ROS at a high rate. ROS production and scavenging are tightly controlled inside cell compartments that differ in their inventory of redox-active proteins present. Those proteins further differ in their coupling to different oxidation and reduction systems.

Classical experimental approaches to address stress responses are frequently based on tissue extraction followed by chromatography- and/or mass spectrometry-based analytical techniques, which make it difficult to separate out individual tissue types, single cells, or even individual subcellular compartments. That has led to a situation in which we still do not systematically understand the diversity of metabolism and physiology between different tissues, cells, and cell compartments in many cases. Genetically encoded biosensors partly address this shortcoming since they are based on fluorescent proteins (FPs) that change their fluorescence properties in response to specific stimuli (Kostyuk et al., 2020). Thus, genetically encoded biosensors allow probing the dynamics of specific metabolites and physiological parameters at high resolution and across all scales of plant structures. The spatial resolution as ensured by precise genetic expression and/or targeting, in combination with dynamic monitoring and adjustable sensitivity, has started to provide detailed insights into plant physiology and introduced shifts in several long-standing paradigms of redox biology and energy metabolism (Meyer et al., 2021).

## Photochemical and enzymatic principles for sensor design

**Figure 1:**
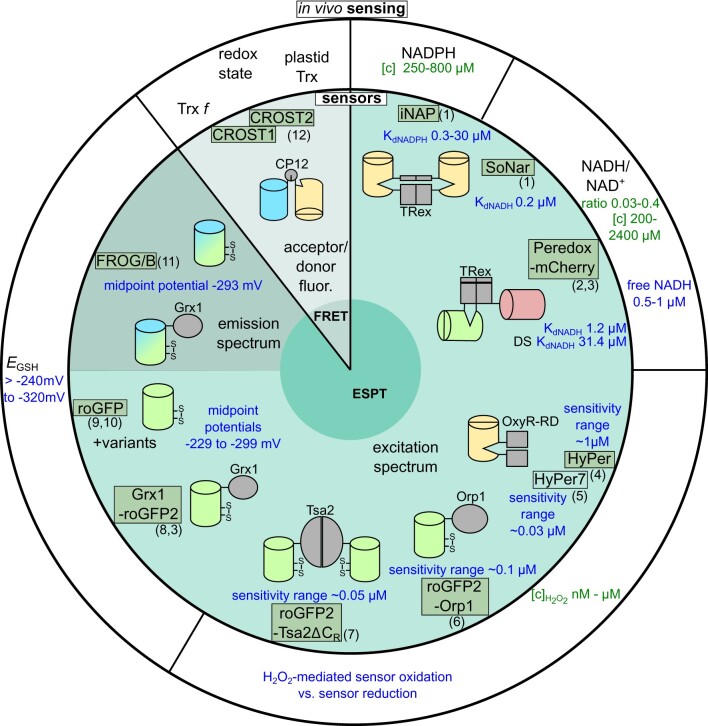
Overview of available sensors for redox-related physiological parameters. Most available redox sensors are designed to exploit excited state proton transfer (ESPT). An exception is the thioredoxin (TRX)-sensor CROST, which exploits Förster resonance energy transfer (FRET) between two fluorescent proteins. While most of these sensors are ratiometric by excitation, the recent addition FROG/B shows a redox-dependent change in its emission properties. The ESPT is influenced by physical constraints on the ß-barrel structure, which are mediated by an inter-strand disulfide in the case of roGFP and FROG/B, or by a fusion partner linked to the N- and C-terminus of a circularly permuted (cp) fluorescent protein (indicated by an indented ß-barrel). Specificity of sensors for their respective analytes is mediated by the fused sensing domain (in gray with names indicated) that specifically binds a ligand substrate (in iNAP, SoNar, Peredox), or specifically forms (HyPer) or transfers a disulfide bond to the sensor (roGFP2-Orp1, roGFP2-Tsa2ΔC_R_). In the FRET-based CROST, part of the TRX-dependent chloroplast protein 12 (CP12) is used as a sensing domain between the FRET-pair. CP12 changes its conformation upon oxidation/reduction and thus leads to a change in energy transfer between the two attached FPs. Names of sensors used in photosynthetic organisms to date are in green boxes, references are given as numbers in brackets: (1) Lim et al. (2020), (2) Steinbeck et al. (2020), (3) Wagner et al. (2019), (4) Costa et al. (2010), (5) Pak et al. (2020), (6) Nietzel et al. (2019), (7) Niemeyer et al. (2020), (8) Gutscher et al. (2008), (9) Meyer et al. (2007), (10) Schwarzländer et al. (2008), (11) Sugiura et al., (2020) and (12) Sugiura et al. (2019). Blue text color shows sensor parameters and physiological parameters estimated based on sensor data. Green text color shows physiological concentrations observed with alternative techniques. Ranges of experimentally observed pool sizes for NADPH^+^/NADP^+^ and NADH^+^/NAD^+^ and their ratios in plants (chloroplasts, mitochondria, cytosol) are from Wigge et al. (1993) and Igamberdiev and Gardeström (2003). Sensitivities towards H_2_O_2_*in vitro* (minimal concentration to elicit detectable response) are from Morgan et al. (2016), Nietzel et al. (2019), and Pak et al. (2020). No absolute H_2_O_2_ concentrations can be measured *in vivo*: sensor redox state is determined by rates of H_2_O_2_-mediated sensor oxidation and sensor reduction mediated by cellular reducing systems (see Box 3). For a review on H_2_O_2_ in plants, see Smirnoff and Arnaud (2019). For midpoint potentials of roGFP variants, see Meyer and Dick (2010).

The functionality of genetically encoded biosensors is based on different physicochemical properties of FPs (Figure 1). To date, several available sensor constructs have been adapted for plants (names in green boxes in Figure 1) and provide insights into the *in vivo* Cys-redox and energy dynamics in plant cells.

### Excitation-based sensors

Dual-wavelength excitation in FP variants is caused by the ionization state of the chromophore that can be shifted between protonated and deprotonated forms. The equilibrium between the two states is governed by an internal hydrogen bond network that permits proton transfer to neighboring amino acids when the chromophore is excited (Brejc et al., 1997; Jung et al., 2005). Interference with the efficiency of this excited state proton transfer (ESPT) through structural changes in the FP ß-barrel leads to changes in excitation properties and hence has opened the door to engineering biosensors for excitation ratio analysis. Sensitivity of the ESPT to the cellular environment can be realized, e.g., by engineering an inter-strand disulfide into the ß-barrel structure of FPs. For a sufficiently strong effect on the ESPT, only two possible positions linking ß-strands 7 and 10 by a disulfide have been identified: C147-C204 and C149-C202. Both disulfides have been exploited in the redox-sensitive yellow fluorescent protein (rxYFP) and the redox-sensitive green fluorescent protein (roGFP) variants 1 to 6 (Ostergaard et al., 2001; Dooley et al., 2004; Hanson et al., 2004). The most commonly used variant to date is roGFP2, which offers photostability, pH-insensitivity (not for emission intensities of the individual excitations, but at the level of the ratio), and a large dynamic range resulting in good signal-to-noise values (Meyer et al., 2007; Schwarzländer et al., 2008; Ugalde et al., 2020). roGFP1 was the first roGFP variant to be reported in a plant context (Jiang et al., 2006) and offers the advantage of a more negative midpoint potential, but it is prone to artifacts by photoconversion (Schwarzländer et al., 2008). roGFP3-6 may offer specialized applications, such as exploration of redox shifts towards more reducing potentials by roGFP3, but although those variants were introduced already in 2004 (Hanson et al., 2004) their use *in plant*a has not yet been reported.

Circular permutation of the FP sequence and fusion of the newly established N- and C-termini to a sensing domain provides another possibility for creating a metastable hydrogen bond network around the chromophore (Baird et al., 1999; Zapata-Hommer and Griesbeck, 2003). Regarding redox-related sensors, circular permuted FPs (cpFPs) have been exploited, e.g., for different variants of the H_2_O_2_-sensitive HyPer-family (Zheng, 1998; Belousov et al., 2006; Pak et al., 2020), the NADH/NAD^+^ sensors Peredox (Hung et al., 2011), SoNar (Zhao et al., 2015) and for the NADPH/NADP^+^ sensor iNAP (Tao et al., 2017; Figure 1). In cpFPs, the newly introduced N- and C-termini leave a cleft in the ß-barrel, which renders the chromophore responsive to conformational changes and concomitantly influences the ESPT. Changes in excitation properties are thus linked to conformational changes induced by substrate-binding to the fused sensing domains. At the same time, the engineered cleft can leave the cpFP chromophore more accessible to protons from the solvent, which introduces the danger of artefactual pH sensitivity from the medium (Schwarzländer et al., 2014).

### Emission-based sensors

In addition to changes in the excitation properties, changes in the ESPT can also lead to changes in emission properties of FPs because the protonated chromophore of GFP emits blue light at excitation while only the deprotonated form fluoresces in green. Further modification of amino acids in proximity to the hydrogen bond network may impact the efficiency of the ESPT and thus causes a clamp of the protonated state of the chromophore and a concomitant change in emission wavelength. This principle has been used to develop the Ca^2+^ sensor protein GEM-GECO1 (Zhao et al., 2011) and, most recently, a newly designed redox-sensitive protein called FROG/B (Sugiura et al., 2020). For the latter, a redox-sensitive probe was created by limiting the efficiency of the ESPT by mutations in combination with an inter-strand disulfide bond linking ß-strands 7 and 10. In this case, the probe is always excited at 400 nm but emits fluorescence predominantly at ∼450 nm (blue) in the reduced state and at ∼510 nm (green) when oxidized.

Förster resonance energy transfer (FRET) between a pair of fluorophores is used in another class of emission-based ratiometric sensors, by linking the FRET pair through a sensing domain that undergoes conformational changes upon oxidation/reduction or substrate binding. This concept of radiation-free energy transfer from a donor FP to an acceptor FP has been used to design the TRX-dependent sensor CROST (Sugiura et al., 2019) and ATeam as a sensor for MgATP^2-^ (Imamura et al., 2009). A similar sensor concept has been implemented in Redoxfluor by integrating a tandem repeat of a redox-sensitive fragment of the yeast transcription factor Yap1 between the FPs Cerulean and Citrine (Yano et al., 2010). This probe has only a low dynamic range and has not yet been used in plants, but exemplifies how further redox-sensitive constructs may be generated in the future. FRET can be detected via several readouts, of which the donor-to-acceptor fluorescence emission intensity ratio has been the most widely used for biosensors, even though fluorescence lifetime imaging (FRET-FLIM) has been gaining popularity in related applications (Xing et al., 2016; Algar et al., 2019).

### Sensor specificity

Sensor specificity is conferred by the fused sensing domain that is dependent on the oxidation and reduction reactions or the binding and dissociation of the ligand, respectively. As the exposed inter-strand disulfide on roGFP and FROG/B is reduced and oxidized preferentially via glutaredoxin (GRX)-mediated catalysis, these sensors equilibrate with the glutathione redox potential (*E*_GSH_) *in vivo* when GRX activity is present (Meyer et al., 2007; Gutscher et al., 2008; Schwarzländer et al., 2008; Sugiura et al., 2020; Figure 1; Box 1). Different variants of roGFPs are available, covering a range of midpoint potentials from –229 mV (roGFP1-iL) to –299 mV (roGFP3; Meyer and Dick, 2010). RoGFP may be used in the free form for sensing of *E*_GSH_ as long as dithiol GRXs (Trnka et al., 2020) are present at sufficient catalytic capacity in the respective compartment (Gutscher et al., 2008). However, in practice roGFP is mainly used as fusion to human Grx1, to ensure comparable kinetic characteristics and circumvent potential problems that may be caused by the abundance and kinetic properties of endogenous GRX in different species, cell types, and subcellular compartments. To date, there are no reports to indicate problems of roGFPs or human Grx1 expression in plants. An exception has been the inability to target Gxr1-roGFP2 to mitochondria that was overcome by an inverted design of the sensor construct (Albrecht et al., 2014). However, mitochondrial roGFP2-Grx1 lines still show a slight developmental phenotype (De Col et al., 2017), and silencing of the sensor has been occasionally observed.

Box 1. The roGFP2 is a sensor for the glutathione redox potential

Glutaredoxins (GRX) catalyze the reversible electron transfer between glutathione (GSH) and roGFP2 (Meyer et al., 2007; Gutscher et al., 2008; Trnka et al., 2020). In the absence of any other known oxidoreductase or thiol oxidase efficiently interacting with roGFP2, this catalyzed bidirectional electron transfer leads to thermodynamic equilibration of the redox potentials *E*_GSH_ and *E*_roGFP2_ described by the respective Nernst equations. The Nernst equations (see Box 1 Figure) include the midpoint potentials of glutathione (–240 mV) and roGFP2 (–280 mV) at pH 7.

Fusion of human Grx1 to either the N- or the C-terminus of roGFP2 guarantees the availability of a functional GRX in close proximity to the engineered disulfide of roGFP2 and improves kinetic responses and reliability of the readouts (Gutscher et al., 2008; Albrecht et al., 2014).

Steady-state measurements in nonstressed wild-type plants have consistently shown that roGFP2 is largely reduced in the cytosol, plastid stroma, the mitochondrial matrix, and peroxisomes (Schwarzländer et al., 2008). With the degree of oxidation (OxD) of roGFP2 approaching 0% under these circumstances, this implies that the probe is well outside its usable dynamic range of 10%–90% OxD. Hence, the redox potential of glutathione (*E*_GSH_) would be –310 mV or even more negative, at pH 7. As the redox potential of thiols is affected by pH, the actual redox potential would be even more negative under more alkaline local pH conditions. *E*_GSH_ necessarily varies with changing amounts of total glutathione and the degree of oxidation of GSSG. Irrespective of the exact physiological concentration, which is in the low millimolar range (Meyer et al., 2001), a redox potential of –310 mV or less implies that the OxD of GSH must be very low leaving effectively only nanomolar amounts of GSSG. This implies GSH:GSSG ratios up to 250.000:1. With this, roGFP2 becomes extremely sensitive to changes in GSSG and thus can be used as a sensor for oxidative events *in planta*. C, cysteine; Chr, chromophore.

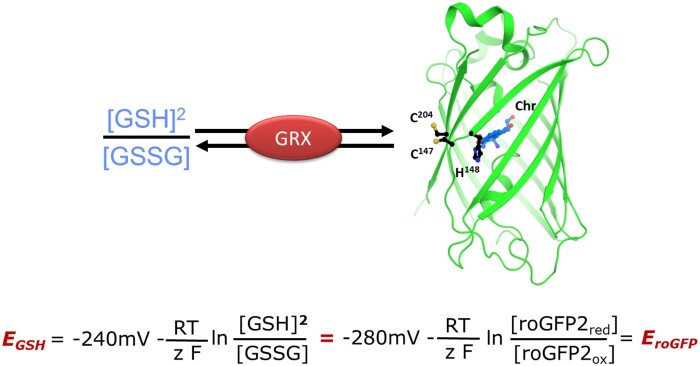

In addition to GRX-mediated oxidation, a different oxidation specificity for roGFP can also be achieved by fusion to different redox-active fusion partners, which may effectively act as thiol oxidase. This strategy has been exploited by fusion of the yeast peroxidase Orp1 (Gutscher et al., 2009) and a mutated version of the 2-Cys peroxiredoxin Tsa2 (Tsa2ΔC_R_) from yeast (Morgan et al., 2016). Both proteins render roGFP2 oxidation particularly sensitive to H_2_O_2_. While roGFP2-Orp1 has been used in Arabidopsis (*Arabidopsis thaliana*; Nietzel et al., 2019; Ugalde et al., 2021), roGFP2-Tsa2ΔC_R_ has been tested in *Chlamydomonas reinhardtii* (Niemeyer et al., 2020). In cpFP- and FRET-based sensors, the specificity depends on the properties of the fused sensing domain, consisting of different well-characterized ligand-binding or redox-sensitive protein domains (Figure 1).

### Assessing sensor characteristics

*In vitro* and *in vivo* sensor responses require careful assessment in terms of specificity, range of sensitivity for the analyte and spectroscopic dynamic range. It is a fundamental – and in most cases justified – assumption that the biochemical and physicochemical sensor behavior is similar *in vitro* and *in vivo*, but this hypothesis requires critical testing for each individual sensor for validation. If possible, the *in vivo* dynamic range should be determined by *in situ* calibration of the sensor, i.e., complete saturation at both ends of the titration curve and compared side-by-side to the *in vitro* calibration. For thiol/disulfide-based redox sensors, this means that full oxidation and reduction need to be achieved as sensor calibration and spectroscopic dynamic range is important for sensor data interpretation and comparability (see Box 2).

Box 2. Sensor calibration and spectroscopic dynamic range

If technically possible, the maximal response of a biosensor in either direction should always be assessed *in vivo*, because cell morphology and particularly differential absorption of either excitation or emission wavelengths bear the risk of causing misinterpretation of sensor data. For a Cys-based redox sensor, membrane-permeable reductants and oxidants can be used, such as dithiothreitol (DTT), diamide, or 2,2′-dipyridyldisulfide (DPS). As an alternative, H_2_O_2_ is often used to achieve full oxidation. However, H_2_O_2_ is not specific for thiols and may cause other damage or interfere with imaging due to formation of O_2_ bubbles released from endogenous activities, such as catalase. The complete *in vivo* reduction (red.) and oxidation (ox.) of the sensor determines its maximal and minimal observable intensity ratios (0% oxidized vs. 100% oxidized) and their difference, the spectroscopic dynamic range (DR, δ; see Figure panels A and B). Dynamic sensor readouts can only carry meaning if the data points of physiological (phys.) measurements are within the DR. When a graphical representation of a ratio readout on a pseudo-color scale is used, the calibration represents the minimal and maximal values of the color scale. After calibration, ratio values can be transformed into degree of oxidation values (OxD; Meyer et al., 2007; Fricker, 2016). As the absolute ratio values can vary depending on the microscopy- or plate reader-setup used, measurements carried out in different laboratories become comparable based on OxD values.

OxD values can be further converted to absolute redox potentials expressed in mV if the midpoint potential (*E*_0_) of the sensor is known (Meyer et al., 2007; Fricker, 2016) (Figure panel C). As redox potentials in mV are dependent on pH (Schwarzländer et al., 2008; Meyer and Dick, 2010), reporting of the assumed compartment-specific pH value is crucial for comparison with literature values. Because each conversion step brings in another level of noise and uncertainty, the log(ratio) is most robust to assess dynamic changes. The near linear range that can be used for meaningful physiological measurements in roGFPs is the midpoint potential of the sensor ±30 mV (equivalent to OxD 10%–90%, Schwarzländer et al., 2008) (Figure panel A)).

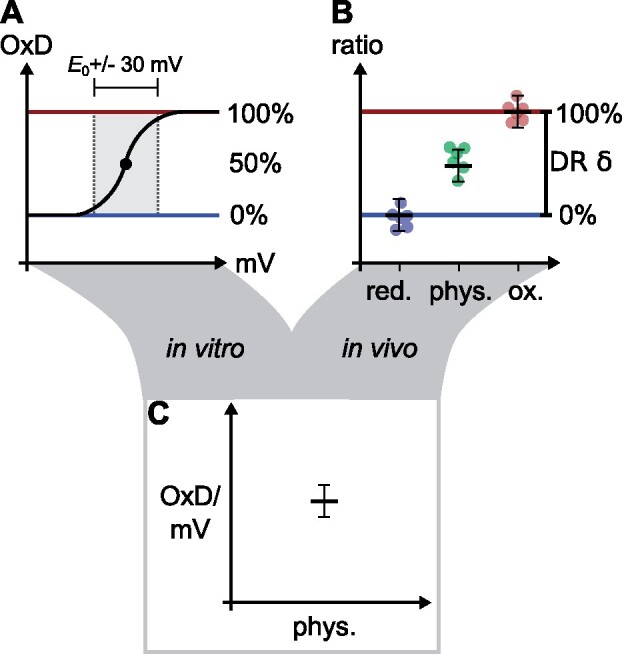

## Live monitoring of plant redox and energy physiology

### H_2_O_2_

Of different chemical compounds generated in the cells that are collectively referred to as ROS, H_2_O_2_ is particularly stable and can diffuse within cell compartments or may be translocated across membranes (Waszczak et al., 2018; Smirnoff and Arnaud, 2019). The local steady-state concentration of H_2_O_2_ depends on the rate of generation and the rate of scavenging. Chemical dyes for H_2_O_2_ are frequently unspecific and provide an accumulative response over time requiring the measurement of their accumulation rate in order to deduce dynamic changes in ROS concentrations (Smirnoff and Arnaud, 2019). In contrast, genetically encoded FP-based probes enable direct access to dynamic information *in vivo*. Local H_2_O_2_ dynamics in response to exogenous H_2_O_2_, H_2_S, light stress, or the elicitation of an immune response have been assessed using HyPer, roGFP2-Tsa2ΔC_R_ or roGFP2-Orp1 probes in the cytosol, the peroxisomes and the mitochondrial matrix as well as the plastid stroma and thylakoid lumen (Costa et al., 2010; Caplan et al., 2015; Exposito-Rodriguez et al., 2017; Scuffi et al., 2018; Nietzel et al., 2019; Niemeyer et al., 2020; Ugalde et al., 2021; Waadt et al., 2020). Key findings include that redox dynamics in different compartments are indeed linked: an elicitor-induced oxidative burst in the apoplast has a time-shifted impact on the cytosolic redox environment (Nietzel et al., 2019, Ugalde et al., 2020). Light stress in chloroplasts triggers redox changes in the cytosol in Arabidopsis and *C. reinhardtii* (Exposito-Rodriguez et al., 2017; Niemeyer et al., 2020; Ugalde et al., 2020a), while a response in the nucleus has only be observed in tobacco epidermis cells (Exposito-Rodriguez et al., 2017). The observations that parallel sensor responses in the stroma and the nucleus were independent of cytosolic ascorbate peroxidase (cAPX) expression levels and that chloroplasts contact nuclei in tobacco epidermis via stromules (Caplan et al., 2015; Exposito-Rodriguez et al., 2017) suggest that membrane contact sites may play a direct role in H_2_O_2_ and/or glutathione exchange between the chloroplast stroma and the nucleoplasm. The dynamic cell-biological linkage between specific organelles represents an intriguing possibility to by-pass high-capacity H_2_O_2_ scavenging systems, as present in the cytosol, to mediate direct inter-compartment ROS-signal transduction. The H_2_O_2_ sensor oxidation steady state is dependent on the rate of oxidation by H_2_O_2_ and the rate of reduction via the endogenous reduction system that the sensor relies on for rereduction in a specific cell compartment (Nietzel et al., 2019; Box 3). In *C. reinhardtii* under light stress, plastid-generated H_2_O_2_ did elicit a sensor response in the cytosol but not in other compartments, suggesting that the cytosol acts as an effective antioxidant barrier (Niemeyer et al., 2020). Notably, weakening the scavenging system in one compartment (such as cytosolic glutathione reductase (GR)) had an impact on sensor responses in other compartments (such as roGFP2-Orp1 in the mitochondrial matrix) in Arabidopsis (Nietzel et al., 2019), indicating a protective role of scavenging capacity in one compartment regarding other subcellular compartments. Thus, the cytosolic response and potential downstream responses in gene expression (such as induction of cAPX; Exposito-Rodriguez et al., 2017) likely depend on local ROS concentrations in different subcellular locations and capacities of several scavenging systems. Differential contributions of different organelles to ROS-related transcriptional events during dark-induced senescence have been reported (Rosenwasser et al., 2011).

Future challenges include the expansion of H_2_O_2_-sensing to additional subcellular compartments and the use of additional sensor variants with properties that match specific physiological requirements (such as the pH-stable HyPer7; Pak et al., 2020). Key questions include which diffusion and transport pathways for H_2_O_2_ originate from different subcellular localizations and how specificity of ROS-signaling is underpinned by the precise spatial and temporal distribution of H_2_O_2_ in the cell. While oxidation of H_2_O_2_-sensors is directly dependent on H_2_O_2_, the subcellular pools of reducing power are also linked, as scavenging and repair enzymes use GSH or TRXs for rereduction. As a consequence, the scavenging of ROS can also result in changes of local *E*_GSH_, especially when ROS generation is higher than the rate of GSSG rereduction or in mutants lacking GR (Nietzel et al., 2019; Box 3).

Box 3. Sensor oxidation and reduction rates *in vivo*

The steady state of sensor oxidation is dependent on oxidation and reduction rates *in vivo* (see Box 3 Figure). For the H_2_O_2_ sensors roGFP2-Orp1 (A) and HyPer family (based on circularly permuted YFP (cpYFP; B) sensors, the oxidation rate is dependent on H_2_O_2_ levels, whereas the reduction rate is dependent on the cellular reducing system resolving the formed disulfide. Both roGFP2 and OxyR-RD (redox domain of hydrogen peroxide-inducible genes activator from *E. coli*) disulfides are reduced by glutathione (GSH) via glutaredoxins (GRX), generating glutathione disulfide (GSSG) (Zheng, 1998; Nietzel et al., 2019). Thus, sensor readouts should be carefully interpreted regarding steady state of sensor oxidation as well as changes in oxidation and reduction rates. For instance, even with specific fusion partners, the rereduction of roGFP2 is dependent on GRXs and the glutathione redox potential *E*_GSH_ and only the relative dynamic change of roGFP2-Orp1 oxidation state over time monitors H_2_O_2_ dynamics (Nietzel et al., 2019).

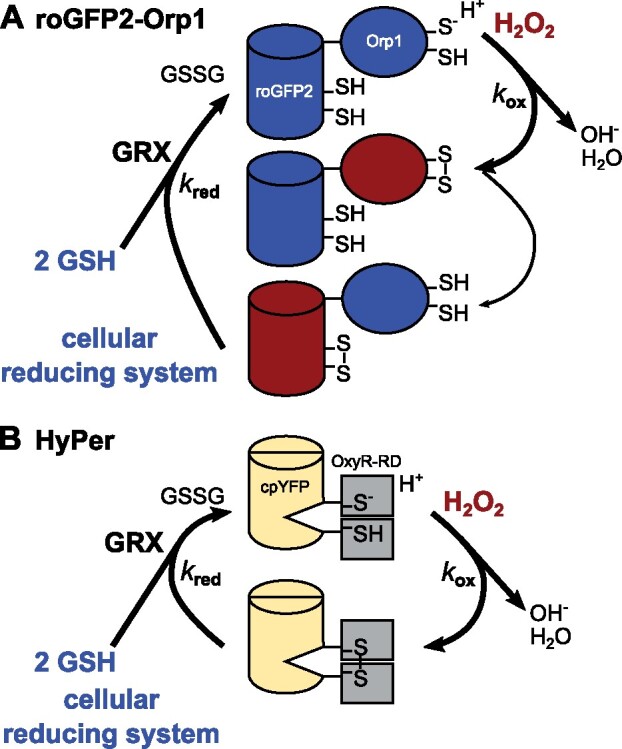

### GSH/GSSG

Glutathione is the most abundant low-molecular-weight thiol in plant cells with concentrations in the low millimolar range (Meyer et al., 2001). Glutathione constitutes an important redox couple in the cell, since two molecules of GSH can be oxidized to form GSSG. The constant cycling between reduction and oxidation by glutathione is most prominently apparent in the glutathione-ascorbate-cycle, in which the regeneration of ascorbate is linked to the oxidation of GSH (Foyer and Halliwell, 1976). Similarly, GSH is also required as an electron donor for glutathione *S*-transferases (GSTs) exhibiting glutathione peroxidase activities for detoxification of organic peroxides and H_2_O_2_ (Dixon et al., 2009). The relative in- and efflux of electrons into and out of the glutathione pool give rise to the steady-state *E*_GSH_. Under non-stress conditions, the maintenance of a high NADPH/NADP^+^ ratio by sufficiently high electron flux into the NADP pool allows GRs to remove GSSG almost completely through reduction to GSH. Early biochemical considerations already led to the conclusion that GR activity would leave only low nM concentrations of GSSG (Veech et al., 1969). Such low amounts of GSSG in the presence of millimolar GSH would lead to highly negative redox potentials of –300 mV and less. While analytical techniques that rely on tissue and cell disruption could not confirm this prediction and found less-reducing *E*_GSH_ values in extracts (Fey et al., 2005; Queval et al., 2008), roGFP2-based measurements in the cytosol, the mitochondria, the peroxisomes, and the plastids of plant cells confirmed a highly reducing *E*_GSH_*in vivo* by observing roGFP2 as almost completely reduced (Meyer et al., 2007; Schwarzländer et al., 2008). With a midpoint potential of –280 mV, the sigmoidal titration curve of roGFP2 allows resolving redox potentials along the near-linear part of the curve (see Boxes 1 and 2). A largely reduced state of roGFP2 hence indicates *E*_GSH_ values of –310 mV (assuming pH 7.0; –340 mV at pH 7.5) or even more negative. In a 2 mM glutathione solution at pH 7.0, a redox potential of –320 mV is established by the presence of only 8 nM GSSG and a GSSG:GSH ratio of 1:250.000. These values are orders of magnitude lower than GSSG:GSH ratios in the order of 1:10 to 1:100 (Mhamdi et al., 2010b; Han et al., 2013; Schnaubelt et al., 2015) typically found in plant tissue extracts. The large difference is caused by two sources of unavoidable experimental errors: (i) mixing of the contents of different cell compartments with different *E*_GSH_ during plant extraction (note that, for instance, ER *E*_GSH_ is much more oxidizing than cytosolic *E*_GSH_; Schwarzländer et al., 2016) and (ii) peroxidative conversion of GSH to GSSG during deproteinization (Hansen and Winther, 2009). While analytical measurement of total GSH and GSSG content may still provide some information about the stress level plants are exposed to, the values can provide only very little information about the physiologically relevant situation in individual compartments and dynamic responses of plant cells to acute stress situations.

The introduction of roGFP-based measurements has allowed capturing true glutathione redox physiology in plants because it enabled monitoring dynamic and local *E*_GSH_ changes in individual subcellular compartments (Meyer et al., 2007; Schwarzländer et al., 2008). RoGFP2 with a midpoint potential of –280 mV has been shown to be the most suitable probe variant to monitor steady-state *E*_GSH_ values and dynamic oxidative changes in response to external triggers. Additionally, roGFP2-based spectroscopic measurements in plant mitochondria at different states of respiratory activity were validated by iodo-TMT-based redox proteomics and quantification of the shift in oxidation of the redox-active Cys-peptides (Nietzel et al., 2020).

Very low total amounts of glutathione or very high degrees of glutathione oxidation are bound to decrease *E*_GSH_ values and would thus drive *E*_roGFP2_ to values outside the linear range of the probe. Due to a lack in GR and oxidative pressure generated by the oxidative protein folding machinery in the secretory pathway, a steep gradient in *E*_GSH_ exists across the ER membrane (Schwarzländer et al., 2008; Meyer et al., 2019). This gradient can be exploited to investigate the topology of membrane proteins by fusing roGFP2 to either the N- or the C-termini and directly observing its orientation towards the cytosol or the lumen (Brach et al., 2009). For dynamic measurements in an environment with a less negative *E*_GSH_, probe variants with less negative midpoint potentials may be used to again match the suitable dynamic range of the probe with the actual *E*_GSH_. A roGFP2 variant, roGFP2iL (midpoint potential –238 mV) has been generated and successfully used in the cytosol of the GSH-deficient mutant *rml1*, which has less than 5% of WT GSH (Aller et al., 2013). The same probe also holds the promise of being suitable for analyses in the ER lumen. Whether roGFP2 variants can be used in the apoplast remains to be tested. The two major caveats in this case may be an acidic pH causing quenching of GFP fluorescence and the lack of reducing power, which is required for roGFPs to attain their oxidation-sensitive form. An interesting approach to circumvent these limitations was recently shown by using bacteria expressing roGFP2 as a biomarker for wash fluids from the surface of flowers and fruits (Liu et al., 2020).

### Glutathione, the cell cycle, and plant development

Gradual depletion of glutathione either pharmacologically through inhibition of biosynthesis or genetically through mutation of the first enzyme in the biosynthetic pathway for GSH, glutamate-cysteine ligase (GSH1), leads to dwarfism or even an almost complete arrest of the cell cycle (Vernoux et al., 2000; Shanmugam et al., 2012). Conversely, genetic restriction of GSH turnover leads to maintenance of GSH even under sulfur deficiency and hence improved root growth under these conditions (Joshi et al., 2019). The apparent link between GSH content and growth has fostered speculations about redox control of the cell cycle. Generally, the cell cycle in eukaryotic cells is synchronized with the metabolic cycle of the cells with S phase and M phase occurring only during the reductive phase of metabolism and G1 in the oxidative phase (Chiu and Dawes, 2012). Based on direct measurements with Grx1-roGFP2, it has been claimed that *E*_GSH_ differs between the cytosol and the nucleus in dividing root cells with *E*_GSH_ in the nucleoplasm being slightly more negative than in the cytosol (Diaz-Vivancos et al., 2015). In non-dividing cells, no such differences have been observed (Schwarzländer et al., 2008; Marty et al., 2009). It is not clear, however, how gradients in *E*_GSH_ across the nuclear envelope could be established because the nuclear pores allow free diffusion of GSH and GSSG and they are large enough to enable the presence of GR in the nucleoplasm (Marty et al., 2009). Redox cycling in synchronized cells, with *E*_GSH_ monitored with Grx1-roGFP2 in both cytosol and nucleus becoming more oxidized during the G1 phase of the cell cycle and more reduced during S- and M-phases, also suggests effective redox coupling between both compartments (de Simone et al., 2017).

Tissue-specific readouts have been observed with sensors for different physiological parameters (De Col et al., 2017; Steinbeck et al., 2020). Similarly, roGFP2 indicates different redox potentials for different stages of flower development in the partially glutathione-deficient mutant *cad2* (García-Quirós et al., 2020). The findings suggest that the flowers of *cad2* mutants have a lower capacity to keep the cellular glutathione pool reduced and maintain high GSH/GSSG ratios but the full biological significance of this observation remains unclear.

### The importance of glutathione reductase

If the total glutathione pool is depleted, the amount of GSSG decreases proportionally because GRs can re-establish the equilibrium between GSH and GSSG (Meyer et al., 2007; García-Quirós et al., 2020). A lack of GR1 in the cytosol causes a shift of *E*_GSH_ towards less negative values by 20–40 mV (Marty et al., 2009). The actual *E*_GSH_ in the cytosol of *gr1* null mutants is thus well within the linear response range of roGFP2 (Box 2) and hence the sensor responds almost instantaneously with a pronounced change in fluorescence to an induced oxidation of the glutathione pool (Marty et al., 2009; Nietzel et al., 2019). With the lack of reductive capacity for removal of GSSG, the respective changes in roGFP2 fluorescence ratios exhibit a larger amplitude and longer duration in the *gr1* background (Nietzel et al., 2019; Ugalde et al., 2020). The fact that *gr1* mutants are fully viable with no or only a very minor phenotype (Marty et al., 2009; Mhamdi et al., 2010a) makes *gr1* a highly suitable model for detection of stress-induced redox signals. This has been exploited in detection of cytosolic oxidation induced by osmotic shock or bacterial elicitors, which could only be detected in *gr1* but not in wild-type plants (Bangash et al., 2019; Nietzel et al., 2019; Ugalde et al., 2020). The reason that *E*_GSH_ in *gr1* does not completely collapse is due to the presence of TRXs and NADPH-dependent TRX reductases (NTRs), which together provide a semi-efficient backup system for GSSG reduction (Marty et al., 2009). The absence of clear cell cycle aberrations in *gr1* despite the less reducing *E*_GSH_ poses question on the significance of changes in *E*_GSH_ of ±30 mV for progression during the cell cycle. These questions have not yet been addressed in more detail and provide a lead for future work.

A second GR isoform, AtGR2 in Arabidopsis (Marty et al., 2019) and PpGR1 in the model moss *Physcomitrium* (Physcomitrella) *patens* (Müller-Schüssele et al., 2020), is dual-targeted to mitochondria and plastids. Deletion of GR2 in Arabidopsis is embryo lethal due to an essential role of GR2 in plastids during very early stages of embryo development well before the photosynthetic apparatus becomes functional (Marty et al., 2019). The cause of lethality is yet to be explored but it is very likely that GSSG accumulates in the stroma with no backup system for reduction or export of GSSG from plastids in place. In mitochondria, GR2 can be deleted because the mitochondrial ABC-transporter ATM3 may export some GSSG (Schaedler et al., 2014). More importantly, however, are the dual-targeted cytosolic-mitochondrial NTRs A and B that, similar to their role in the cytosol, are responsible for reducing GSSG together with mitochondrial TRXs (Marty et al., 2019). Despite this reductive power acting on GSSG, roGFP2-Grx1 targeted to the matrix of mitochondria lacking GR2 indicates a less reducing *E*_GSH_ resulting in a pronounced *E*_GSH_ gradient across the mitochondrial envelope. The lack of GR2 in the mitochondrial matrix is associated with enhanced respiration and deregulated TCA cycle dynamics, which suggests a decreased resource efficiency of energy metabolism (Nietzel et al., 2020). In pronounced contrast to Arabidopsis, the lack of stromal GR in *P. patens* does not cause lethality (Müller-Schüssele et al., 2020). Grx1-roGFP2 measurements clearly indicate a pronounced oxidation in the stroma, which results in decreasing photosynthetic efficiency with increasing light intensities.

### Light dependency of *E*_GSH_ in plastids

Probing *E*_GSH_ in different photosynthetic species has revealed that the stromal *E*_GSH_ is not as stable as in other cell compartments, but responds to changes in light conditions and to light/dark and dark/light transitions in a remarkably dynamic manner (Haber et al., 2021; Hipsch et al., 2020; Müller-Schüssele et al., 2020; Sugiura et al., 2020). FROG/B was used to probe light-dependent *E*_GSH_ dynamics in vegetative cells and heterocysts in Anabaena and showed *E*_GSH_-dependent reduction of the sensor in the light and oxidation in the dark (Sugiura et al., 2020). In plants, *E*_GSH_ responded dynamically within seconds and a similar range (changes of about 30 mV) to light and darkness in *P. patens* (Müller-Schüssele et al., 2020). These dynamic changes occurred in the presence of a GR and could, in theory, be based on changes in GSSG or GSH levels with rapid changes of the low levels of GSSG being the more likely candidate. Notably, stromal *E*_GSH_ dynamics were recently also reported in a diurnal light and dark regime in Arabidopsis (Haber et al., 2021), pointing to a general underlying mechanism leading to an oxidative shift of stromal *E*_GSH_ in the dark. The biological relevance of stromal *E*_GSH_ dynamics requires further investigation to unravel cause-and-effect relationships and to test the potential consequences for photosynthetic function and regulation.

### Thioredoxin

Reducing power for cellular thiol-based scavenging and repair systems stems from either GSH or TRXs. Reducing equivalents such as NADH and NADPH (see the following paragraph) or electrons from photosynthetic electron transport are used by different reductases to regulate the oxidation state of TRXs (Geigenberger et al., 2017). Electron flux through the light reactions is directly linked to metabolic flux through the photosynthetic carbon reactions via TRX-operated thiol switches. Thus, the dynamic monitoring of TRX redox state is highly desirable to establish an *in vivo* understanding of photosynthetic regulation but was to date mainly realized by Western blotting of protein extracts (Yoshida et al., 2014) or *in vitro* assays with reconstituted redox relays between different proteins (Yoshida and Hisabori, 2017). Recently, two different TRX redox sensors were constructed. Trx1-rxRFP is an intensiometric TRX sensor based on the fusion of a circularly permuted redox-sensitive RFP to human cytoplasmic Trx1 (Fan et al., 2017). While Trx1-rxRFP has not yet been successfully used in plants, the FRET-based biosensor CROST was generated specifically for use in photosynthetic organisms, using the stromal TRX target protein CP12 from Arabidopsis (Sugiura et al., 2019). CP12 is specific to photosynthetic organisms and undergoes disulfide-based conformational changes upon oxidation/reduction, forming a complex with the two CBB cycle enzymes glyceraldehyde-3-phosphate dehydrogenase (GAPDH) and phosphoribulokinase (PRK) in its oxidized form (Wedel et al., 1997). In the GAPDH/CP12/PRK complex, the CBB cycle enzymes are inactive and protected from oxidation but can be rapidly reactivated via TRX f and m isoforms (Marri et al., 2009; Marri et al., 2014). Sugiura and colleagues (Sugiura et al., 2019) used a partial sequence containing one disulfide of CP12 from two different species (*A. thaliana* CP12-2 in CROST1 and the cyanobacterium *Anabaena* spec. CP12 in CROST2) to create a FRET-based ratiometric sensor linking a variant of mTurquoise and cp173-mVenus as FRET pair. While the sensor response of CROST1 was specific to AtTRX-f1 *in vitro*, CROST2 interacted with TRX f, m, x, y, z from several tested species. As the ratio of cpmVenus/mTurquoise in CROST sensors was shown to be responsive to pH-changes (decrease of cpmVenus fluorescence with lower pH) in the physiological range of the plastid stroma (Sugiura et al., 2019), the sensor response *in vivo* will be influenced by pH changes by a decrease of dynamic range. However, *in vivo* experiments in *A. thaliana* leaf chloroplasts confirmed a decrease of FRET in the light that was dependent on electron flux through the light reactions and an increase of FRET in the dark in the range of 10 min. The *in vivo* dynamic sensor responses of CROST1 and CROST2 confirm the dynamics of CBB redox regulation already described by biochemical and gas-exchange methods (Stitt et al., 1989; Yoshida et al., 2014). CROST sensors are thus a promising starting point to further dissect TRX-dependent redox dynamics *in vivo* in different photosynthetic model species.

### Reducing equivalents and ATP

Cellular physiology can only be upheld by the biosynthesis of ATP to maintain a high adenylate charge that in turn drives many metabolic and biosynthetic enzymes. Similarly, reducing equivalents of appropriate potential in the form of NAD(P)H are needed to maintain metabolism and redox homeostasis in an aerobic environment. In plants, phosphorylation of ADP and P_i_ to ATP is driven by chemiosmosis in the mitochondria and in chloroplasts in the light. Photosynthetic light reactions additionally reduce NADP^+^ to NADPH, while NAD^+^ is reduced to NADH mainly in catabolism through glycolysis and the TCA cycle. Thus, ATP (adenylate charge), NADPH/NADP^+^, and NADH/NAD^+^ levels differ between the different compartments, depending on light conditions and metabolic mode (Stitt et al., 1982; Wigge et al., 1993; Igamberdiev and Gardeström, 2003; Gardeström and Igamberdiev, 2016). However, the transfer of reducing equivalents and phosphorylation potential between compartments is crucial and relies on metabolite shuttles such as the malate valve (Selinski and Scheibe, 2018) or the triose phosphate shuttle, or direct transport of ATP and ADP via ADP/ATP carriers (Hoefnagel et al., 1998; Haferkamp et al., 2011).

The FRET-based ATeam sensor consisting of CFP and YFP fused to the epsilon subunit of ATP synthase from *Bacillus subtilis* as sensing domain for MgATP^2-^ (Imamura et al., 2009; Kotera et al., 2010) has already been used in plants (Hatsugai et al., 2012; De Col et al., 2017; Voon et al., 2018, Elsässer et al., 2020). With the help of the sensor-variant ATeam 1.03-nD/nA, mitochondria were confirmed as the major suppliers of ATP to the cytosol using mitochondrial inhibitors and monitoring cytosolic sensor readout, as well as in an *ex situ* assay using isolated mitochondria (De Col et al., 2017). Dynamic and tissue-specific energy charge of the cytosol was pinpointed by a lower level of MgATP^2-^ in non-green tissues, a decrease after wounding and under low oxygen conditions (De Col et al., 2017; Wagner et al., 2019) as well as by monitoring the restart of mitochondrial function during seed imbibition and germination (Nietzel et al., 2020). MgATP^2-^ concentration in the plastid stroma was found to be lower than in the cytosol and responsive to light, with both pools being independent of each other, except for young seedlings where ATP is imported efficiently into the stroma via chloroplast nucleotide transporters (Voon et al., 2018; Elsässer et al., 2020).

It needs to be emphasized that ATeam is specific for MgATP^2-^ concentration, which is the bioactive form of ATP for most enzymes, but not adenylate charge (i.e. the ratio between ATP, ADP, and AMP). That means that changes in energetic status can only be inferred under the assumption that the total size of the adenylate pool remains stable. That assumption is justified for rapid transitions, such as onset hypoxia within minutes; by contrast, different MgATP^2-^ concentrations between different cells, tissues, or even plants lines may indicate either different adenylate charge or different adenylate pool sizes (or a combination of both). Further the MgATP^2-^-complex requires sufficient Mg^2+^ availability meaning that the sensor may be an attractive option to probe for intracellular Mg^2+^ (rather than ATP) changes at limiting Mg^2+^ availability. To measure ATP/ADP, other sensor variants have been engineered, which show significant pH sensitivity, however, and their use has not been reported for plants so far (Berg et al., 2009; Tantama et al., 2013).

Sensing of reducing equivalents has become accessible through the *Thermus aquaticus* Rex (T-Rex) as NADH/NAD^+^ sensing module that has been used to design the cpFP-based sensors Peredox and SoNar (Hung et al., 2011; Zhao et al., 2015) as well as the NADPH sensing SoNar variant iNAP (see Figure 1; Tao et al., 2017). Recently, all three sensor types have been used to probe metabolic flexibility and the linkage of NAD(P)H pools between different compartments (Elsässer et al., 2020; Lim et al., 2020; Steinbeck et al., 2020). Experiments with different light/dark regimes in combination with chemical inhibition of light reactions, respiration, or photorespiration revealed tight and dynamic metabolic coupling between cytosol and organelles. After illumination, stromal NADPH concentration and NADH/NAD^+^ ratio increased, with a concomitant increase in cytosolic NADH/NAD^+^ ratios. These observations confirm the functionality of reducing equivalent interconversion and export to the cytosol via metabolite shuttles (e.g. malate/oxaloacetate; Elsässer et al., 2020). Cytosolic NADH/NAD^+^ ratios were also influenced by catabolic activities: sucrose feeding increased NADH/NAD^+^ ratios by fueling glycolysis; blocking mitochondrial respiration by inhibitors or low oxygen increased NADH/NAD^+^ ratios, revealing mitochondria as governors of cytosolic NAD redox homeostasis (Steinbeck et al., 2020).

Similar to cytosolic MgATP^2-^ levels, imaging of whole Arabidopsis seedlings revealed differences in NADH/NAD^+^ ratios between cells and tissues, most strikingly between shoot and root, with root cells exhibiting particularly high NADH/NAD^+^ ratios (Steinbeck et al., 2020). At etiolation, the NAD pool in root cells showed a much more oxidized state, indicating profound metabolic reprogramming associated with photomorphogenesis even in the non-aerial organs.

These studies initialized the *in vivo* imaging of ATP and NAD(P) redox status in plant cells and have already revealed important principles of the *in vivo* dynamics of metabolic coupling of chloroplasts and mitochondria to the cytosol. This will pave the way for further studies of how photosynthetic and respiratory status influences the whole plant physiology. Surprisingly, leaf cytosolic NADH/NAD^+^ ratios increased transiently and markedly in response to elicitor-exposure (Steinbeck et al., 2020), indicating that *in vivo* biosensing allows the exploration of previously unknown modes of crosstalk between central redox metabolism and plant immunity.

## Limits and improvement of sensors

### Sensor design/*in vitro* limits

Whether a genetically encoded biosensor is suitable to address a specific biological question needs to be carefully assessed in each individual case considering the inherent biochemical and physicochemical characteristics of the sensor.

- The thermodynamic behavior of a sensor is determined by the midpoint potential of the redox-sensitive disulfide as well as the ability to interact with other redox-active proteins. For sensors that do not react with, but reversibly bind the analyte, the equivalent to midpoint potential is the binding/dissociation curve as determined by K_m_ and Hill coefficient (measure of cooperativity when more than one substrate-binding site is present at a sensor).
- Binding and dissociation kinetics of ligands determine the kinetic behavior. For the roGFP-based sensors, the relative kinetics of the sensor with different interaction partners (GRX, TRX, H_2_O_2_, etc.) sets the specificity of the sensor (Meyer et al., 2007; Nietzel et al., 2019). In addition, the achievable temporal resolution of *in vivo* measurements is limited by oxidation/reduction rates or on- and off-rates of analyte binding *in vivo*. While those rates are typically fast and allow detection of physiological changes in the order of seconds or even milliseconds, binding and dissociation rates may be limiting for measuring rapid NAD redox dynamics using Peredox (Steinbeck et al., 2020).
- The spectroscopic dynamic range of a sensor designates its maximal possible spectral response, usually determined in an *in vitro* calibration (Box 2) on purified sensor protein and sets the limits for sensor use. It is important to note that the dynamic range is wavelength-specific meaning that different experimental approaches that use slightly different excitation and/or emission windows will deliver different maximal spectroscopic dynamic ranges (Ugalde et al., 2021).
- Sensor characteristics should match the expected range/changes of the coupled redox pool in the used *in vivo* system (see Figure 1). For instance, *E*_GSH_ in the plant cytosol leads to nearly complete roGFP2 reduction, limiting its responsiveness to reduction of the *E*_GSH_ and the monitoring of reductive changes. To cover the *E*_GSH_ range that is represented in different cell compartments, several roGFP variants have been engineered with different midpoint potentials (Meyer and Dick, 2010; Aller et al., 2013). However, there is currently no sensor with a midpoint potential to match the most reducing (e.g. cytosol; *E*_GSH_ ∼320 mV) and most oxidizing (e.g. ER ∼208 mV) cell compartments (Schwarzländer et al., 2016). Development of such sensor variants will be desirable for fully dynamic measurements in both directions (oxidation and reductions) in the future. Analogously, analyte-binding sensors, such as the NADH/NAD^+^ sensor Peredox, can become saturated if their affinity is too high in the context of the physiological concentrations of the bound compounds, NAD^+^ and NADH. Although the affinity of Peredox has turned out well-matched with the physiological NAD^+^ and NADH concentrations in the cytosol of Arabidopsis leaves, protein engineering of the ligand-binding pocket by targeted mutagenesis has allowed to adjust the measurement range for situations of highly reduced NAD pools (as observed in root tissues or after specific metabolic stimuli) by lowering NADH binding affinity (Peredox-mCherry DS; Steinbeck et al., 2020). For iNap affinity variants have been generated to cover a large physiological range of NADPH (Tao et al., 2017), and in recent work different affinity variants have been selected as suitable for dynamic measurements in the cytosol (iNap1) and the chloroplast stroma and peroxisomes (iNap4) of Arabidopsis cotyledons (Lim et al., 2020). Since the exact compartment-specific analyte concentrations are often not known or only rough estimations exist based on analytical approaches, empirical *in vivo* testing of different sensor variants is generally advisable.
- Biosensors may respond to different parameters, in addition to the desired parameter, and if those parameters change, *in vivo* artifacts can arise. A particularly widespread additional sensitivity is that to pH. *In vitro* assessment is required to estimate robustness of sensor responses. Most GFP-variants are quenched at low pH and circularly permuted fluorescent proteins, in particular, can be responsive to pH changes in the physiological range, due to direct protonation of the exposed chromophore (Schwarzländer et al., 2014). This behavior can lead to misinterpretation of sensor responses *in vivo*, and – if present – deserves appropriate caution. pH-dependence as a side-specificity in biosensors must be suppressed or compensated for, or carefully considered when interpreting the measurement results. Significant efforts have been made to address this problem and multiple different approaches have been chosen.

The readout of roGFP2 is pH-insensitive, because the pH sensitivity of roGFP2 fluorescence is proportional across its spectrum and is canceled out by ratioing two excitation wavelengths. For HyPer sensors, a corresponding H_2_O_2_-insensitive Cys mutant, SypHer (Poburko et al., 2011), needs to be used as parallel pH control while in HyPer7 pH-dependence has been largely overcome by mutagenesis, resulting in pH-robustness of the ratiometric readout (Pak et al., 2020). The pH sensitivity of SoNar and iNap sensors has been addressed by using a control construct that cannot bind to the nucleotides and allows pH correction (Zhao et al., 2015; Tao et al., 2017). The strategy of pH correction has recently also been adopted in plants (Lim et al., 2020). Since a control construct is only available for one of the sensor affinity variants and the correction is only valid for the nucleotide-free state of the sensor, it remains unclear what degree of accuracy the pH correction can achieve. In the case of Peredox, the cpFP variant cpT-Sapphire with a low pKa was chosen and the initial pH-sensitive sensor sequence was mutagenized, and a pH-insensitive variant was selected largely circumventing the danger of pH artifacts (Hung et al., 2011; Steinbeck et al., 2020).

Applying those corrections is critical in plants where metabolic changes often coincide with pH changes (Behera et al., 2018; Wagner et al., 2019; Lim et al., 2020). However, due to identical spectroscopic properties of the control constructs, the control measurements can only be carried out in separate biological material, which increases required sample sizes and may average out physiological meaningful responses. Co-expressing a pH sensor of different colors in the same sample is possible and allows more refined correction and has recently been done for HyPer in tobacco leaves using the pH sensor pHRed (Exposito-Rodriguez et al., 2017). Yet it is rare that both sensors share identical pH responses (spectroscopic dynamic range, pKa) and hence the accurate correction remains technically demanding. Hence, if available, pH-inert sensor variants are generally preferable for *in planta* analyses.

### Limits to meaningful biosensor usage *in planta*

Sensor responses *in vivo* can differ from the sensor responses monitored *in vitro*. An *in vivo* calibration (Box 2) can determine the *in vivo* dynamic range that may be affected by interfering autofluorescence, chromatic artifacts, or interactions with the local (protein) environment. While disulfide-based sensors can be calibrated by membrane-permeable oxidants and reductants, the *in vivo* calibration of metabolite sensors is often not feasible. Metabolite levels, such as MgATP^2-^, cannot be easily driven to the necessary extremes in all living plant tissues, and the feasibility of permeabilization of the plasma membrane to apply saturating concentrations externally, like often employed in mammalian cells or yeast, remains to be tested case by case (Loro et al., 2016). If an *in vivo* calibration is not possible because the necessary values lie outside the experimentally reachable range in cells (such as for NAD(P)H/NAD(P)^+^, MgATP^2-^ or pH) the *in vitro* calibration of purified sensor protein and/or calibration of the sensor in plant tissue extracts (as recently done for pH calibration by Wagner et al., 2019) can be taken as an empirical reference point for the sensor response *in vivo*.

In addition, sensor concentrations cannot be controlled *in vivo*, which introduces challenges regarding data normalization and comparability of intensiometric sensor data, e.g., between different cells or treatments. Sensors exhibiting changes into opposite directions in either the excitation or the emission properties or sensors containing two different fluorophores enable ratiometric analysis as a prerequisite for a robust and quantitative *in vivo* readout. Ratiometric sensor behavior comes with the advantage of internal normalization, i.e., the signal ratio is independent of the amount of sensor (expression level, field of view) and this principle applies to both chemical dyes and protein-based sensors (Grynkiewicz et al., 1985; Remington, 2011). In Peredox-mCherry, an engineered domain consisting of circular-permuted tSapphire and two NAD(H)-binding TRex domains responds to the NADH/NAD^+^-ratio via changes in emission intensity without a change in the opposite direction in another emission range (intensiometric sensor; Figure 1). Here, the fusion of the intensiometric sensor module to mCherry enables a ratiometric readout (Hung et al., 2011). Along the same rationale, a versatile Matryoshka-platform for an indifferent reference FP with a long emission wavelength nested in a circular permuted reporter FP with the respective sensor domain has been developed (Ast et al., 2017).

Importantly, sensor use in plants is also limited by the ability to target sensors to all subcellular compartments. While it has often been speculated that sensor-analyte interaction may affect local cell physiology (which is unavoidable to a degree and indeed appears to account for growth phenotypes of Arabidopsis lines expressing specific hormone biosensors in the cytosol; Waadt et al., 2014), the observation of similar biosensor-related phenotypes independent of sensor specificity has raised the hypothesis that the expression of large organelle-targeted fusion protein constructs may compromise organelle import (De Col et al., 2017). Thus, either transgenic lines with developmental phenotypes, such as dwarfism as for mitochondrial ATeam (De Col et al., 2017), or no transgenic lines were obtained, such as for mitochondrial Peredox-mCherry (Steinbeck et al., 2020), mitochondrial and peroxisomal SoNar as well as mitochondrial iNAP (Lim et al., 2020). This illustrates the necessity to carefully document arising phenotypes of transgenic fluorescent sensor lines.

Similarly, redox sensors are not yet available for all compartments. To date, dynamic apoplastic redox sensing is of high interest to plant development, signaling, and stress responses (Franck et al., 2018; Smirnoff and Arnaud, 2019), but no apoplastic redox sensor line is available yet. While this is not a problem of protein targeting, prerequisites for apoplastic redox sensing are robustness of fluorescence signal towards low pH and a sufficiently oxidizing midpoint potential to match apoplastic physiology. Further, dynamic Cys-based redox sensing in the apoplast would rely on the availability of an extracellular reducing system for Cys, the existence of which is unknown to date.

There are additional technical limitations when working with plant-based systems, e.g., combining the readout of sensor fluorescence with illumination regimes to monitor plant physiology under active photosynthesis. To date, this problem has been solved by using custom illumination setups for confocal microscopes or camera-based systems (Voon et al., 2018; Sugiura et al., 2019; Elsässer et al., 2020; Haber et al., 2021; Hipsch et al., 2020; Lim et al., 2020; Müller-Schüssele et al., 2020; Steinbeck et al., 2020) or circumvented by fixing the sensor redox state using cell-permeable thiol-modifying agents (Niemeyer et al., 2020).

A general consideration for the interpretation of biosensor data is that they can only capture the steady state of a parameter, and not the in- and efflux that set this steady state. For instance, a stable MgATP^2-^ concentration may be detected, despite MgATP^2-^ being generated and consumed at high rate. Steady-state changes can only be observed through biosensing if the balance between generation and consumption is shifted and it remains generally unknown whether a change in the steady state is due to a change in the influx, the efflux, or both. In the case of redox sensors, this means that electron flux cannot be measured. Redox sensors, such as those from the roGFP or HyPer families, that are based on the specific oxidation of Cys via a fusion partner, the steady-state oxidation level is read out, which is dependent on both, the rate of oxidation and the rate of rereduction. Thus, the specific oxidation and the capacity of the linked reduction systems (e.g. TRX or GRX/*E*_GSH_) determine the sensor steady-state oxidation level *in vivo* (Box 3).

## Concluding remarks

The use of genetically encoded biosensors in plants has already allowed for the observation of hitherto unknown dynamics and the specification of links between redox pools previously know or hypothesized based on data from complementary techniques. This area of plant research is currently in an explorative phase, driving development of new hypotheses and concepts on plant metabolic flexibility and information transfer between subcellular compartments (see Outstanding Questions). After fundamental questions into the characteristics of the sensors applied to plants are answered and the first conclusions regarding the dynamics of energy metabolism of plants are drawn, future reverse genetics studies in transgenic sensor lines will give insights into what molecules are transferred between compartments and how. Further, expansion of sensor uses to more model species and crops is starting. This development and the increasing technical possibilities to gain systemic overview of plant responses to environmental challenges in whole plant camera-based systems (Fichman et al., 2019; Haber et al., 2021; Zandalinas et al., 2020) will certainly pave the way to use biosensors on an organismic level. While sensor use is limited by sensor characteristics and the fact that only a low number of fluorescent readouts can be combined at the same time in the same cell, the field is developing towards combining multiple sensors (Kostyuk et al., 2018; Waadt et al., 2020) and towards *in vivo* multiplexing of physiological parameters (Nietzel et al., 2019; Wagner et al., 2019), opening the field of systemic dynamic sensing of plant physiology.

**OUTSTANDING QUESTIONS**

- Which redox pools are linked between subcellular compartments?
- How dynamic is this linkage in changing environmental/metabolic conditions?
- Precisely which molecules are transferred between compartments, and how?
- How is this additional layer of communication between cell compartments integrated with known signaling pathways?
- What is the interplay between redox pools in different tissues, and at the whole plant level?

## Notes

S.J.M.-S., M.S., and A.J.M. wrote the manuscript.

The author responsible for distribution of materials integral to the findings presented in this article in accordance with the policy described in the Instructions for Authors (https://academic.oup.com/plphys/pages/general-instructions) is: Stefanie J. Müller-Schüssele (stefanie.mueller@uni-bonn.de).
